# Bobble-Head Doll Syndrome Caused by Aqueductal Web–Associated Obstructive Hydrocephalus in an Adult: A Case Report with Video

**DOI:** 10.5334/tohm.1246

**Published:** 2026-10-08

**Authors:** Rina Shibayama, Toshiyuki Enomoto, Dai Kawano, Hiromasa Kobayashi, Takashi Morishita, Koichiro Takemoto, Hiroshi Abe

**Affiliations:** 1 Department of Neurosurgery, Faculty of Medicine, Fukuoka University 7-45-1, Nanakuma, Jonan-ku, Fukuoka 814-0180, Japan

**Keywords:** Bobble-head doll syndrome, no-no tremor, hydrocephalus, aqueductal web

## Abstract

**Background:**

Bobble-head doll syndrome (BHDS) is a rare movement disorder usually observed in children and associated with third ventricular lesions or hydrocephalus.

**Case report:**

A 24-year-old man presented with progressive memory impairment, gait instability, urinary incontinence, and irregular no-no head tremor with a sensory trick. MRI revealed severe obstructive hydrocephalus due to an aqueductal web. Ventriculoperitoneal shunting was performed, and the tremor resolved within one week. Neurological symptoms improved, and he remained independent 18 months after surgery.

**Discussion:**

Adult BHDS is extremely rare. Early recognition and appropriate CSF diversion can provide favorable outcomes.

## Introduction

Bobble-head doll syndrome (BHDS) is a rare hyperkinetic movement disorder characterized by rhythmic head movements, typically occurring at a frequency of approximately 2–3 Hz [1]. The movements are most commonly of the anterior–posterior (yes–yes) or lateral (no–no) type. BHDS is frequently associated with lesions around the third ventricle, including suprasellar arachnoid cysts, third ventricular cysts, and obstructive hydrocephalus [2].

Most reported cases occur in early childhood, typically before five years of age, and adult onset is extremely rare [3]. The pathophysiological mechanism remains unclear, although compression of structures around the third ventricle, including the basal ganglia and midbrain tegmentum, has been proposed [4].

We report a rare adult case of BHDS associated with aqueductal obstruction caused by an aqueductal web. The tremor resolved rapidly after ventriculoperitoneal shunt placement. Pre- and postoperative videos demonstrate the characteristic movement disorder and its resolution.

## Case report

A 24-year-old man with no history of developmental delay had been living independently and working after graduating from university. Nine months before presentation he began working in another prefecture but returned to his hometown shortly afterward due to progressive malaise.

Approximately four months before presentation he developed involuntary head movements, memory impairment, gait instability, and urinary incontinence. He visited our hospital for evaluation.

On admission, he was disoriented with a Glasgow Coma Scale score of 14 (E4V4M6). Neuropsychological testing revealed cognitive impairment with a Montreal Cognitive Assessment score of 12/30, Mini-Mental State Examination score of 16/30, and Hasegawa Dementia Scale-Revised score of 13/30.

Neurological examination demonstrated involuntary head movements in the sitting position. The movements consisted of irregular lateral oscillations consistent with no-no tremor. The tremor amplitude and frequency were variable. The patient could not voluntarily suppress the tremor; however, the movements temporarily stopped when his hand touched his face or head, suggesting the presence of a sensory trick. Although he could stand with assistance, severe postural instability was present and independent ambulation was impossible (Video 1). The patient exhibited a mildly wide-based gait with irregular cadence, veering from a straight line, and marked unsteadiness during turning, suggesting truncal ataxia. No definite appendicular ataxia, dysarthria, or pyramidal signs were identified. Eye movements were not systematically evaluated at the initial examination.

**Video 1:** **Preoperative head tremor**. Irregular no-no head tremor observed in the sitting position. The tremor could not be voluntarily suppressed but transiently ceased when the patient touched his face or head, suggesting a sensory trick. Marked postural instability is also evident. Speech was clear without apparent dysarthria.

Magnetic resonance imaging revealed marked enlargement of the lateral and third ventricles. A membranous structure consistent with an aqueductal web was observed within the cerebral aqueduct, causing obstructive hydrocephalus. In addition, an old lesion of uncertain etiology was identified in the right temporal lobe. Although a previous hemorrhagic event could not be excluded, its relationship to the aqueductal obstruction remained unclear.

Heavily T2-weighted imaging demonstrated that the floor of the third ventricle was indistinct, making the anatomical relationship with the basilar artery unclear (Figure 1). Therefore, endoscopic third ventriculostomy was considered potentially unsafe, and ventriculoperitoneal shunting was selected.

**Figure 1:**
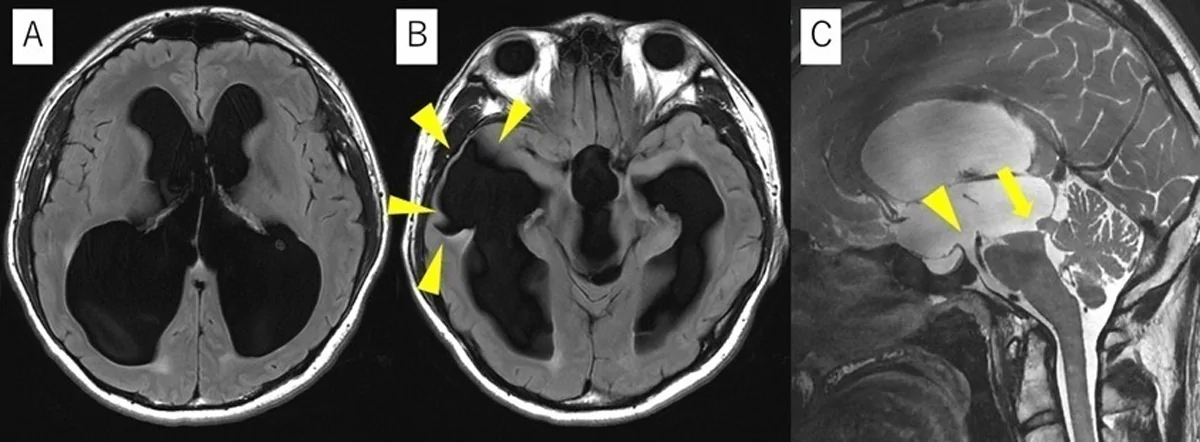
Preoperative magnetic resonance imaging. **(A)** Axial fluid-attenuated inversion recovery (FLAIR) image demonstrating marked enlargement of the lateral ventricles. **(B)** Axial FLAIR image showing an old lesion in the right temporal lobe adjacent to the inferior horn of the lateral ventricle (arrowheads). **(C)** Sagittal heavily T2-weighted image demonstrating an indistinct floor of the third ventricle with an unclear anatomical relationship between the third ventricular floor and the basilar artery (arrowheads). An aqueductal web is also identified at the entrance of the cerebral aqueduct (arrow).

A programmable shunt system (Codman Certas Plus® programmable valve (Codman Certas Plus® programmable valve; Codman & Shurtleff, Inc., Raynham, MA, USA)) was placed with an initial valve setting of 6 (180 mmH_2_O).

Within one week after surgery, the head tremor completely disappeared. Cognitive impairment, gait disturbance, and urinary incontinence gradually improved (Video 2).

**Video 2:** **Postoperative outcome**. Complete resolution of head tremor within one week after shunt placement. Cognitive and gait function gradually improved. At 16-months follow-up, improved gait is maintained with mild residual postural instability.

Two months after surgery, imaging revealed enlargement of the subdural space with development of a subdural hematoma (Figure 2). Because the patient was asymptomatic, the valve setting was increased to 7 (215 mmH_2_O) and the patient was managed conservatively. The hematoma subsequently resolved.

**Figure 2:**
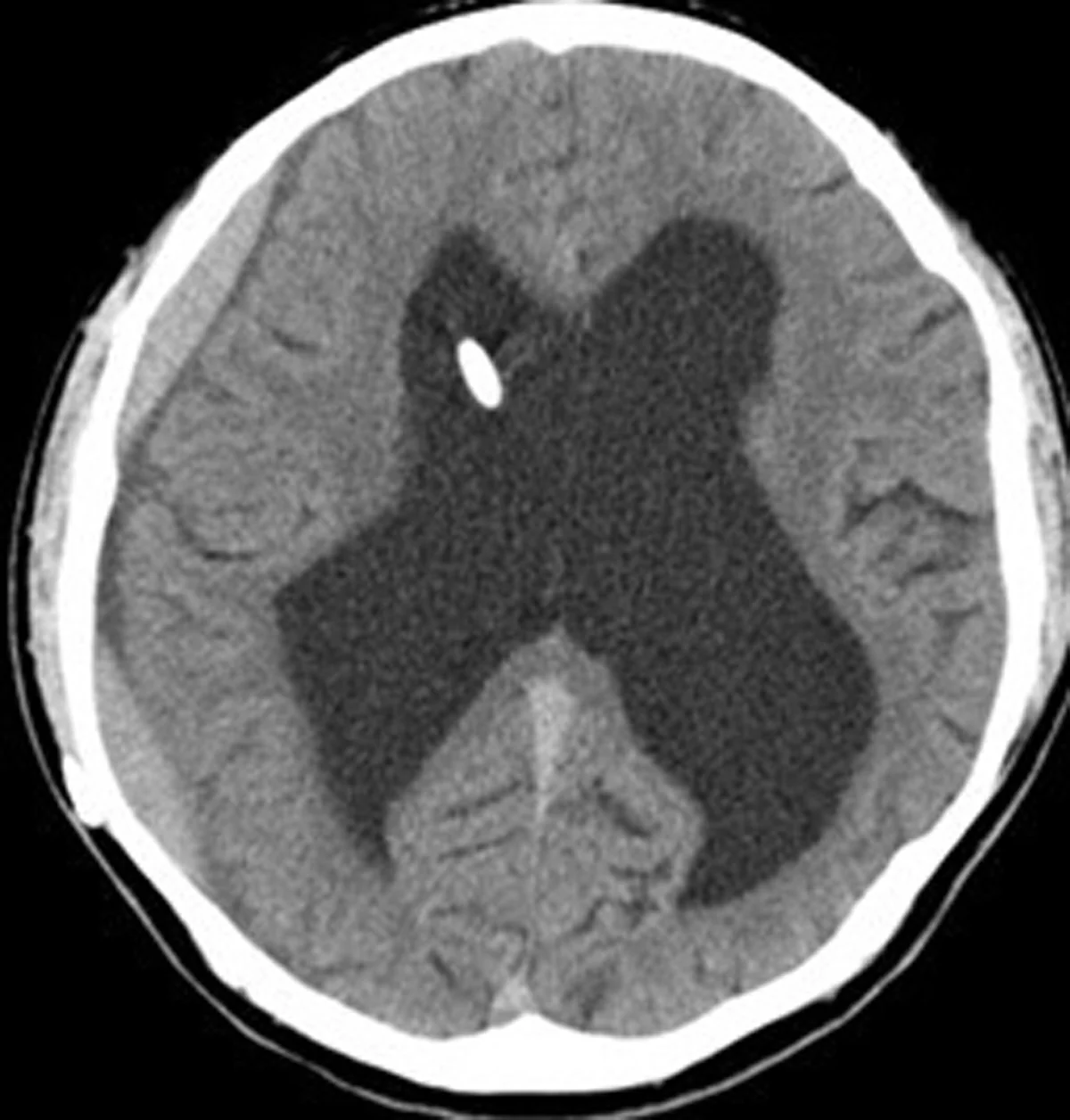
Postoperative computed tomography. Axial image demonstrating placement of the ventricular catheter in the right frontal horn. A subdural hematoma is observed beneath the inner table of the right cranial vault.

At the strong request of the patient and his family, the valve setting was returned to 6 (180 mmH_2_O) ten months after surgery. Follow-up imaging has demonstrated gradual ventricular reduction without recurrence of the subdural hematoma.

Although mild memory impairment persists and he has not returned to work, the patient remains independent in activities of daily living 18 months after surgery.

## Discussion

BHDS is a rare movement disorder characterized by rhythmic head movements occurring at a frequency of approximately 2–3 Hz [5]. The movements may be of the yes–yes type, no–no type, or a mixed pattern. Lesions around the third ventricle, including suprasellar arachnoid cysts, third ventricular cysts, and obstructive hydrocephalus, are the most commonly reported underlying causes [3].

The exact mechanism of BHDS remains uncertain. Several hypotheses have been proposed [3]. One theory suggests that ventricular enlargement compresses the basal ganglia and thalamus, leading to abnormal neuronal activity within extrapyramidal circuits and resulting in dystonic head movements. Another hypothesis proposes that distortion of the dentato-rubral-thalamic pathway caused by compression of the midbrain tegmentum or red nucleus produces cerebellar tremor.

In addition, some authors have suggested that repetitive head movements may represent a learned behavior that transiently alters cerebrospinal fluid dynamics and reduces intracranial pressure [1].

A review of the literature identified 10 previously reported cases of BHDS associated with aqueductal obstruction (Table 1) [1,3,6,7,8,9,10,11,12]. Including the present case, a total of 11 cases were analyzed. The reported age at onset ranged from 4 months to 14 years, making the present case unusual because of adult onset. Among these cases, yes–yes tremor was observed in six cases, no–no tremor in four cases, and a mixed pattern in one case. Etiologies included six cases of aqueductal stenosis, two cases of aqueductal web, one trapped fourth ventricle, one pineal region tumor, and one tectal tumor.

**Table 1:** **Clinical characteristics, suppression factors, and surgical outcomes of 11 cases of bobble-head doll syndrome (BHDS) associated with aqueductal obstruction or stenosis**.


CASE (YEAR)	AGE	CAUSE	DIRECTION OF BOBBING	VOLUNTARILY SUPPRESS	SUPPRESSION FACTORS/SENSORY TRICK	ATAXIA/INTENTION TREMOR	TREATMENT	POSTOPERATIVE COURSE

1. Nellhaus (1967) [6]	10y	aqueductal stenosis	No–No	yes	sleep	yes/no	Pudenz valve shunt	Resolved within 8 months

2. Russo (1974) [7]	1y	aqueductal stenosis	Yes–Yes	yes	sleep	no/no	VA shunt	Resolved after 8 days

3. Tomasovic (1) (1975) [8]	10m	aqueductal stenosis	Yes–Yes	no	sleep	yes/no	VP shunt	Markedly decreased within 4 months

4. Tomasovic (2) (1975) [8]	5.5y	aqueductal stenosis	Yes–Yes	yes	sleep, recumbent position	yes/yes	VP shunt	Resolved within several days

5. Deonna (1976) [9]	1.5y	aqueductal stenosis	No–No	no	sleep, supine position	no/no	Torkildsen procedure	Resolved

6. Jensen (Carrea) (1978) [10]	11m	aqueductal stenosis	Yes–Yes	N/A	N/A	N/A	Shunt surgery	No change

7. Coker (1986) [11]	6m	Trapped 4V	Yes–Yes	yes	sleep, supine position, concentration	no/no	4V shunt	Resolved, then returned after 2 mos, then resolved after revision

8. Bhattacharyya (2003) [1]	13y	aqueductal web	Yes–Yes	yes	sleep, concentration	yes/yes	VP shunt	Resolved

9. Bhattacharyya (2014) [12]	11y	Pineal mass	Mixed	yes	sleep, concentration	yes/no	VP shunt	Immediate resolution

10. Renne (1) (2018) [3]	14y	Tectal tumor	No–No	no	sleep, supine position	N/A/no	ETV	Immediate resolution in recovery room

11. Our case	24y	aqueductal web	No–No	no	sleep, concentration, touching the cheek	yes/no	VP shunt	Resolved after 7 days


This table summarizes the clinical profiles of 11 cases where BHDS was secondary to aqueductal lesions (obstruction, stenosis, or tumors). It details the primary direction of head movements, the presence of voluntary suppression, and specific suppression factors (sensory tricks) such as mental concentration or postural changes. Associated neurological signs, including truncal ataxia and intention tremor, are also recorded. The “Postoperative Course” column highlights the efficacy and immediacy of surgical interventions, including ventriculoperitoneal (VP) shunting and endoscopic third ventriculostomy (ETV).

Truncal ataxia was reported in 6 of the 11 cases, and 2 of these patients also exhibited intention tremor. Interestingly, both patients with intention tremor had yes–yes head movements, suggesting that cerebellar dysfunction may contribute to the clinical manifestations of BHDS in at least a subset of patients. In the present case, re-evaluation of the videos demonstrated several features suggestive of dystonia, including a sensory trick, mild retrocollis, left laterocollis, elevation of the left shoulder, and dystonic posturing of the distal upper extremities during walking. However, the patient also exhibited a mildly wide-based gait, irregular cadence, veering from a straight line, and marked instability during turning, consistent with truncal and gait ataxia, whereas no definite appendicular ataxia was identified. These findings suggest that both dystonic and cerebellar mechanisms contributed to the clinical manifestations in the present patient. Importantly, these mechanisms are not mutually exclusive. Compression of the enlarged third ventricle and midbrain may affect both the basal ganglia–thalamic circuitry and cerebellar outflow pathways, including the dentato-rubro-thalamic tract. Furthermore, recent evidence has demonstrated microstructural abnormalities of the dentato-rubro-thalamic tract in patients with cervical dystonia, supporting the concept that cerebellar network dysfunction contributes to the pathophysiology of dystonia [13]. Therefore, the clinical manifestations of BHDS may reflect overlapping dysfunction of basal ganglia and cerebellar motor networks rather than isolated impairment of a single motor circuit.

Aqueductal web is a rare cause of obstructive hydrocephalus characterized by a thin membranous obstruction within the cerebral aqueduct. It may be congenital or acquired secondary to inflammation, infection, or hemorrhage, although the etiology often remains uncertain. While aqueductal web usually presents during infancy or childhood, incomplete obstruction may allow symptoms to remain compensated until adolescence or adulthood. A similar case of BHDS associated with aqueductal web has previously been reported by Bhattacharyya et al. [1]. The unusually late presentation in the present patient may likewise reflect long-standing but initially compensated obstruction. BHDS associated with aqueductal web remains extremely rare, and recognition of this condition is important because appropriate cerebrospinal fluid diversion can result in rapid symptom resolution.

With regard to treatment, 8 cases were managed with shunt surgery and 1 with endoscopic third ventriculostomy (ETV), while older cases were treated with procedures such as Pudenz valve placement or the Torkildsen procedure. In 10 of the 11 cases, surgical treatment resulted in improvement or complete resolution of symptoms, indicating that surgical intervention is highly effective for BHDS associated with obstructive hydrocephalus. The treatment of third ventricular hydrocephalus has evolved over time, and both ventriculoperitoneal shunting and ETV are currently widely used. Although the success rates of these procedures are considered comparable, shunt surgery is associated with a higher incidence of complications such as obstruction, malfunction, and subdural fluid collection [14]. Therefore, ETV is generally preferred when anatomically feasible.

In the present case, however, the anatomical relationship between the floor of the third ventricle and the basilar artery was unclear, and ventriculoperitoneal shunting was considered safer than ETV. Postoperatively, the patient developed subdural hematoma associated with enlargement of the subdural space. This finding suggests that in cases of chronic hydrocephalus presenting with BHDS, careful management of shunt pressure—potentially at a higher setting than that used for normal-pressure hydrocephalus—and regular radiological follow-up are necessary.

## Conclusion

We report a rare adult case of bobble-head doll syndrome caused by third ventricular hydrocephalus due to aqueductal web. The clinical manifestations of BHDS may reflect both dystonic and cerebellar mechanisms, which are likely to coexist rather than represent mutually exclusive pathophysiological processes.

Surgical treatment, including ventriculoperitoneal shunting or endoscopic third ventriculostomy, is effective for symptom resolution. When shunt surgery is selected, careful attention should be paid to the risk of subdural fluid collection, and a relatively higher pressure setting may be preferable.

The precise pathophysiological mechanism of BHDS remains unclear, and further accumulation of cases is required to better understand this rare condition.
